# Polycrystallization effects on the nanoscale electrical properties of high-k dielectrics

**DOI:** 10.1186/1556-276X-6-108

**Published:** 2011-01-31

**Authors:** Mario Lanza, Vanessa Iglesias, Marc Porti, Montse Nafria, Xavier Aymerich

**Affiliations:** 1 Dept. Eng. Electrònica, Edifici Q, Campus UAB, 08193 Bellaterra, Spain

## Abstract

In this study, atomic force microscopy-related techniques have been used to investigate, at the nanoscale, how the polycrystallization of an Al_2_O_3_-based gate stack, after a thermal annealing process, affects the variability of its electrical properties. The impact of an electrical stress on the electrical conduction and the charge trapping of amorphous and polycrystalline Al_2_O_3 _layers have been also analyzed.

## Introduction

To reduce the excess of gate leakage currents in metal-oxide-semiconductor (MOS) devices, the ultra thin SiO_2 _gate oxide is replaced by other high-k dielectric materials [1]. However, high-k-based devices still show some drawbacks, and therefore to have a better knowledge of their properties and to improve their performance, a detailed electrical characterization is required. Many researches have been devoted to the study of the electrical characteristics of high-k gate dielectrics, mainly using standard wafer level characterization techniques on fully processed MOS capacitors or transistors [1-4]. However, since the lateral dimensions of complementary MOS devices are shrinking to a few tens of nanometers or below, for a detailed and profound characterization, advanced methods with a large lateral resolution are required. In this direction, conductive atomic force microscope (CAFM), as demonstrated for SiO_2 _and other insulators [5-14], is a very promising tool which allows for a nanometer-resolved characterization of the electrical and topographical properties of the gate oxide. Characterization at the nanoscale allows us to study which factors determine the electrical properties of the dielectric stack, and details on how they affect them. For example, some manufacturing processes (such as high-temperature annealing) can alter their electrical properties because of the polycrystallization of the high-k dielectric, which can affect its electrical homogeneity [15]. Recently, the CAFM has been started to be used to evaluate the electrical conduction of polycrystalline high-k dielectrics. While in some polycrystalline materials the electrical conduction seems to be mainly related to the bulk of grains [16], in others, current can flow preferentially through grain boundaries (GBs) [17-20]. Since this topic can be crucial for the successful inclusion of high-k dielectrics in electron devices, in this study, AFM-related techniques have been used to investigate, at the nanoscale, the effect of the high-k material polycrystallization (derived from an annealing process) on the conductivity and charge trapping of Al_2_O_3_-based stacks for Flash memories.

## Experimental

Gate stacks, which consist of a nominal 10-nm-thick Al_2_O_3 _layer and a 1-nm-thick SiO_2 _interface layer on top of a p-type Silicon substrate, have been analyzed. After the Al_2_O_3 _deposition, some of the samples were annealed by rapid thermal process (RTP) in nitrogen at 750 or at 950°C. The electrical properties of the stack were measured using a Dimension 3100 AFM provided with CAFM and Kelvin probe force microscope (KPFM) modules. The CAFM allows us to obtain, simultaneously to the topography, current images of the structures, by means of applying a constant voltage between the tip and the sample during a surface scan, and *I*-*V *characteristics on fixed locations, by means of applying ramped voltage tests. The KPFM allows us to obtain, simultaneously to the topography, images of the contact potential difference (CPD) between the tip and the substrate. For all the current and CPD measurements, Si tips with a Pt-Ir or diamond coating were used. Topographic images have been obtained in tapping mode using Silicon ultra sharp tips without coating, which offer a better spatial resolution. Other techniques such as transmission electron microscopy (TEM) and X-ray reflectometry have been also used to perform a physical analysis of the structures.

### As-grown dielectrics

**Figure 1:**
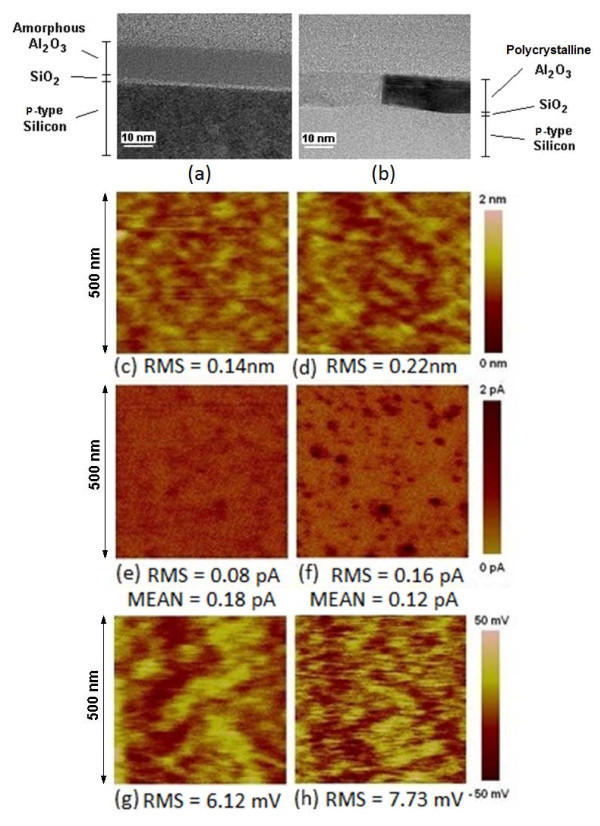
**TEM images (a, b), topographic maps (c, d), current maps (e, f), and CPD maps (g, h) for amorphous (left column) and polycrystalline (right column) samples**. The values of the most relevant parameters are shown.

To begin with, a physical analysis of the two samples has been performed with TEM and X-ray Reflectometry. Figure 1 shows cross-sectional TEM images of the sample annealed at (a) 750 and (b) 950°C. Note that the different layers of the stack structure are clearly distinguished (SiO_2 _interfacial layer and high-k dielectric). Moreover, it can also be observed that the sample annealed at 950°C shows a polycrystalline structure (the different gray intensities in the high-k layer corresponding to the different orientations of the nanocrystals), while the sample annealed at 750°C remains amorphous. These results were confirmed from GIXRD measurements [21]. From TEM images, the crystalline grains seem to have a diameter of 15-30 nm. The surface of the two samples has also been studied from AFM topography maps. Figure 1 shows topographic images obtained on the (c) amorphous and (d) polycrystalline structures. The root mean square (rms) value of the images is also included. Although in this experiment the resolution of the set-up does not allow to distinguish single crystals, the figure indicates an increase of the surface roughness after polycrystallization, in agreement with [11,22]. Finally, since a thermal annealing process can also affect the thickness of the layers of the stack, the actual physical thicknesses of the SiO_2 _and Al_2_O_3 _films were determined from X-ray Reflectometry (Table 1). Note that, after polycrystallization, a reduction in the thickness of the high-k layer is observed [23], leading to a smaller equivalent oxide thickness (EOT) [17].

**Table 1:** Thicknesses of the Al_2_O_3 _and SiO_2 _layers obtained from X-ray reflectrometry on the amorphous and polycrystalline samples

Phase	**Al**_**2**_**O**_**3 **_**thickness (nm)**	**SiO**_**2 **_**thickness (nm)**	EOT (nm)
Amorphous	14.6	1.0	7.3
Polycrystalline	12.4	1.2	6.6

The EOT was estimated by considering a permittivity of 9.1 for the Al_2_O_3 _layer.

The impact of the polycrystallization of the Al_2_O_3 _layer on the electrical conduction of the gate stack has been analyzed at the nanoscale from current and CPD images obtained on fresh structures (*before *an electrical stress). Figure 1e,f shows two current images obtained on the amorphous and polycrystalline sample, respectively, at 10.25 V (their average and rms values are included in the figure). Note that smaller currents (in average) are measured in the polycrystalline stack. However, since the EOT of the polycrystalline sample is smaller (see Table 1), its lower conductivity can only be attributed to the crystallinity of the stack and not to the reduction of the oxide thickness. Figure 1f also shows that the rms value of the current and, therefore, the electrical inhomogeneity of the polycrystalline stack is larger. Both samples have also been analyzed with KPFM [21], which can provide information about the presence of charge and trapping centers in the stack. Figure 1 shows two CPD images obtained on the amorphous (g) and polycrystalline sample (h). Their rms value is also included. Again, after crystallization, the deviation increases, suggesting larger inhomogeneities in its trapping properties.

**Figure 2:**
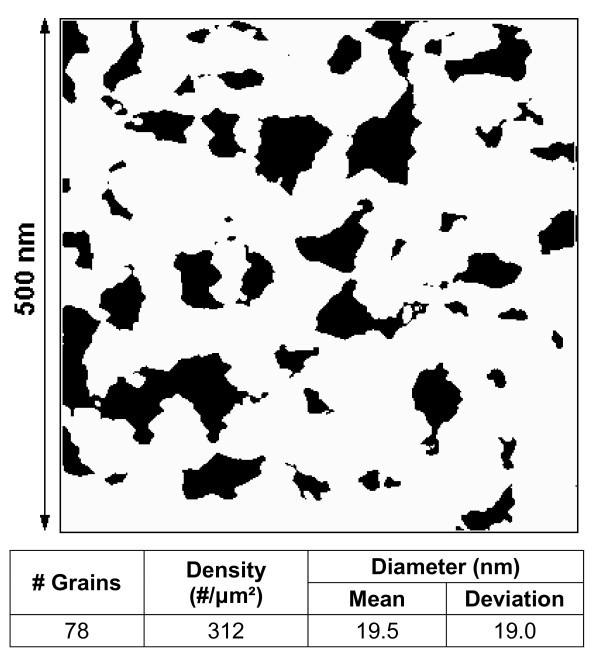
**Grain analysis of a current image measured on the sample annealed at 950°C**. White areas correspond to currents above the noise level (0.2 pA.)

The results presented until now demonstrate that the polycrystallization of the Al_2_O_3 _layers leads to a larger inhomogeneity of the sample conduction and charge trapped in the stack, which could be attributed to the different electrical properties of nanocrystals and grain boundaries. Taking advantage of the large lateral resolution of the CAFM, a more detailed analysis has been performed to explore this point. Toward this aim, the areas with smaller conductivity have been evaluated from the current images of the sample that has polycrystallized (Figure 2). In Figure 2, the white areas correspond to the regions with a current above 0.2 pA, while the black areas show a current lower than the noise level. The table in Figure 2 includes the results of the statistical analysis of the image, indicating the number, density, and size of the regions with a smaller conductivity (black regions). Note that the average size of these areas is approximately 20 nm, which is compatible with the results obtained from TEM images (Figure 1) for the sizes of the Al_2_O_3 _nanocrystals. Therefore, these results suggest that the regions with a smaller conductivity could be related to the grains in the polycrystalline structure: the nanocrystals are more insulating whereas the grain boundaries show a larger conductivity. Note that, in Figure 2, the width of the regions attributed to the grain boundaries is much larger than that estimated in other studies [22], when AFM measurements were performed in ultra high vacuum (UHV). This apparent discrepancy can be explained by considering the impoverishment of the lateral resolution of CAFM experiments when working in air, compared to UHV measurements [22,24]. The differences in electrical behaviors between nanocrystals and grain boundaries could explain the larger inhomogeneity detected in the current and CPD images after polycrystallization. Grain boundaries, probably with an excess of some kind of defects or trapping sites generated during the polycrystallization (which could be related to O-vacancies [25]), could enhance the gate current through them, probably because of trap-assisted-tunneling (TAT) through the defects detected with KPFM [23].

It is important to emphasize that the correlation of the leaky positions with the grain boundaries is a qualitative result, since the resolution in these experiments is not high enough to resolve grain boundaries. This is because the CAFM measurements presented in this section have been performed with Si tips coated with a metallic layer in ambient conditions, drastically reducing its lateral resolution to approximately 20 nm [26]. Note, however, that other experiments, with sufficient resolution, have shown the relation between leaky sites and grain boundaries [27,28]. The section "Influence of the environment on the resolution of grain boundaries" will be devoted to investigate how the CAFM resolution can be improved.

### Stressed dielectrics

**Figure 3:**
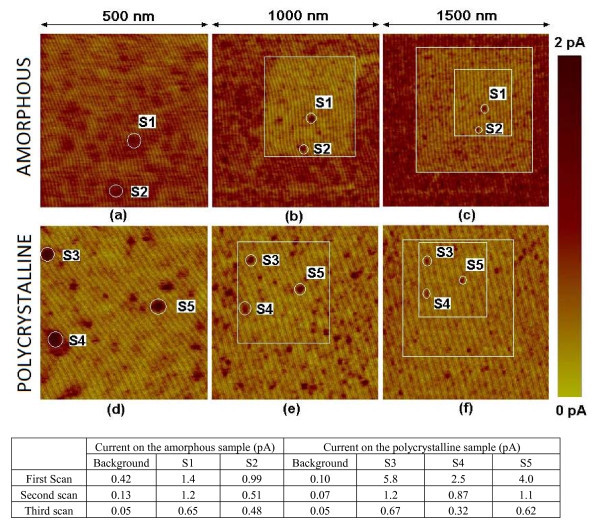
**First scans (a, b) and two consecutive zooms out (b/e, c/f) on amorphous (a, b, c) and polycrystalline (d, e, f) samples**. Their sizes are (a, d) 500 nm × 500 nm (b, e) 1 μm × 1 μm and (c, f) 1.5 μm × 1.5 μm. The applied voltage was 11.5 V in all cases. The table shows the evolution of the maximum current driven by the spots S1-S5 and on background areas for both samples.

In this section, the impact of an electrical stress on the electrical conduction and charge trapping of the Al_2_O_3 _layers will be analyzed at the nanoscale. Differences between amorphous and polycrystalline structures will be evaluated. First, the effect of the degradation (*before *breakdown) induced during a constant voltage scan on a certain area of the oxide will be investigated. Toward this aim, sequences of current images have been collected, on amorphous and polycrystalline Al_2_O_3 _samples. First, a 500 nm × 500 nm area was scanned by applying a large enough constant voltage to induce degradation. Afterward, two zoom-outs were done, and larger areas were scanned (1000 × 1000 nm^2 ^and 1500 × 1500 nm^2^), which included the previously scanned smaller areas. Figure 3 shows a sequence of three images measured on the amorphous (a, b, and c) and polycrystalline (d, e, and f) samples. The first scan corresponds to images (a) and (d) and, the last scan, to images (c) and (f). The sizes of the images are (a and d) 500 nm × 500 nm, (b and e) 1 μm × 1 μm, and (c and f) 1.5 μm × 1.5 μm. The applied voltage was 11.5 V in all the cases. This procedure allows us to compare areas that have been subjected to different stresses--or, in other words--areas that have experienced different degradation levels.

Comparing Figure 3a,d, which corresponds to the first image (fresh area) measured on amorphous and polycrystalline structures, respectively, results similar to those shown in the previous section are obtained. On polycrystalline samples, background conduction is smaller (table of Figure 3, first scan). However, the leaky sites of polycrystalline structures (spot S3, S4, and S5) have a larger conductivity compared to those of amorphous samples (S1 and S2). As discussed in the previous section, the larger current differences in the polycrystalline structure could be attributed to the differences in the conductivities between the crystals (background) and grain boundaries (leaky sites).

The effect of the stress has been analyzed from the images measured during the zoom-outs. On the amorphous sample (Figure 3b), the central area (which was previously pre-stressed, Figure 3a) shows smaller currents than the rest of the scanned region. A similar behavior can be observed in Figure 3c, where three concentric areas can be distinguished: a first central area with the smallest current value (three scans), another second area with a larger current (two scans), and the peripheral and the most conductive area (only one scan, that is, a fresh area). The quantitative values of the background current on the three concentric areas are shown in the table of Figure 3. In the amorphous sample, the background current decreases significantly as the stress proceeds, making the structure less conductive. This behavior, as already pointed out for SiO_2 _layers [29] or high-k dielectrics, can be related to negative charge trapping in the native defects or in traps generated during the stress. In the case of polycrystalline structures (Figure 3d,e,f and table), the decrease in the background conductivity is less important when compared to amorphous samples. This result suggests a smaller impact of the stress at the positions where crystals are present.

Although, in polycrystalline samples, regions with background currents (which can be probably related to positions with a crystal under the CAFM tip) seem to be more resistive and robust to an electrical stress than amorphous oxides, this behavior cannot be extended to the weak spots (leaky sites). As an example, the table in Figure 3 shows the maximum current in different spots and its evolution with the stress on amorphous (spots S1 and S2) and polycrystalline samples (S3, S4, and S5). Note that the weak spots in the polycrystalline structure show, before the stress ("first scan"), larger leakage currents compared with the amorphous gate oxide. However, after the stress ("second scan" and "third scan"), the reduction of current through these spots is larger than those in amorphous structures. Therefore, initially, the leaky sites of polycrystalline samples are, from an electrical point of view, weaker (their conductivity is higher) than those in amorphous oxides (because the dielectric is thinner or because of the presence of defects that enhance tunneling). However, as the stress proceeds, a larger amount of charge is trapped in the as-grown or generated defects on the leaky positions, leading to a higher reduction of the current compared to amorphous oxides. Since these leaky regions could be related to the grain boundaries between nanocrystals, charge trapping (in as-grown or generated defects) mainly occurs at those locations, leading to a higher reduction of the conductivity compared to the background areas. In amorphous samples, no distinction between crystals and grain boundaries can be observed, and so trapping is randomly distributed in the gate area.

**Figure 4:**
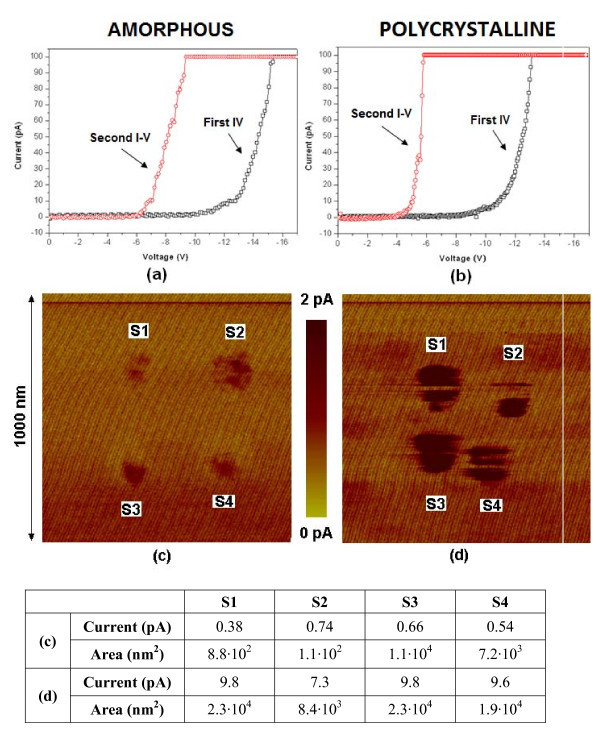
**Current images obtained on an amorphous (c) and polycrystalline (d) sample where previously, four RVS where applied to induce BD**. The voltage applied during the scan was, in both cases, -8.6 V. **(a, b) **correspond to typical *I*-*V *curves measured on those positions. The maximum current and area of the BD spots can be found in the table.

Finally, the impact of *breakdown *(BD) was also investigated on polycrystalline and amorphous oxides. Toward this aim, first, ramped voltage stresses (RVS) with a current limit of 100 pA and with the same ending voltage have been applied on different oxide locations until BD. Figure 4a,b shows an example of two consecutive *I*-*V *curves measured on an amorphous and a polycrystalline structure, respectively. Note that, in the second RVS, current can be measured at much lower voltages in both cases, which is an indication that BD has been triggered. Moreover, the voltage at which current is measured in the polycrystalline structure is lower, pointing out a harder BD. After the measurement of the *I*-*V *curves, current images of the areas that contain the stressed locations have been collected. Figure 4c,d shows the current images obtained on a 1 μm × 1 μm area of the amorphous (c) and the polycrystalline (d) sample where four RVS had been previously applied until BD at different locations. Regions with larger currents are observed, which correspond to the BD spots. The table in the figure shows the maximum current and area of the BD spots generated on each sample. Note that, for the amorphous sample, the BD areas are smaller and the post-BD electrical conduction is lower, suggesting softer BD events, in agreement with the post-BD *I*-*V *curves. From these results and those obtained during the degradation stage, it seems reasonable to speculate that, in polycrystalline structures (with harder BD), BD takes place at the weaker regions, that is, the grain boundaries. Therefore, the presence of grain boundaries on Al_2_O_3 _layers could reduce significantly the reliability of MOS devices due to their lower robustness.

### Influence of the environment on the resolution of grain boundaries

Some authors have suggested that, when working with a CAFM in air, the tip-sample contact area increases, probably due to the presence of a water layer on the sample (and, therefore, a water meniscus between the tip and the surface), which can reduce the lateral resolution of the measurements [19,30,31]. Since the grain boundaries width is in the range of few nanometers [32,33] and the CAFM lateral resolution in air when using metal coated tips is about 10 nm, grain boundaries could not always be resolved. This would explain why in the sections "As-grown dielectrics" and "Stressed dielectrics" when working with a CAFM in ambient conditions, a point-to-point correlation between the topographical and electrical properties (in particular, between the leaky sites and the grain boundaries position) was not possible. For this reason, when a higher resolution is needed, CAFM in vacuum or UHV has been used [12,24,34,35]. In this section, the impact of environmental conditions on the CAFM electrical resolution for the study of polycrystalline structures will be analyzed.

**Figure 5:**
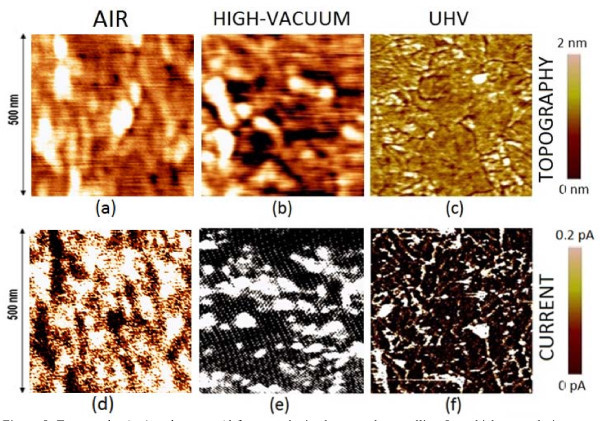
**Topography (a, b, c) and current (d, e, f) maps obtained on a polycrystalline 5-nm-thick HfO_2 _sample in different environments: air (a, d), high-vacuum (b, e), and ultra-high-vacuum (UHV)--(c, f)**. In current images, white areas correspond to regions with a current above 0.2 pA, while the black areas show a current lower than the noise level.

Toward the above aim, topographical and current images obtained on polycrystalline high-k dielectrics at different ambient conditions have been compared. Figure 5 shows topographical (first row) and current (second row) maps measured on a 5-nm-thick HfO_2 _layer grown on a Si substrate, obtained in air (a and d), high-vacuum (b and e, 1.2 × 10^-6 ^mbar), and UHV (c and f, 10^-9 ^mbar) [35]. In current images, the white areas correspond to the regions with a current above 0.2 pA, while the black areas show a current lower than the noise level. Note that as pressure decreases (and, therefore, the size of the water meniscus is reduced), topography images show a better-defined granular structure, which can be attributed to single (or a cluster of) nanocrystals (grain boundaries would correspond to the depressed regions [32]). Current maps show a similar behavior. While in UHV, a clear granular pattern can be observed [35] (which overlaps with that observed in the topographical image, indicating that current flows mainly through grain boundaries, as suggested in the sections "As-grown dielectrics" and "Stressed dielectrics"), in HV and, specially, in air, the granular structure is not so clearly distinguishable and a point-to-point correlation of the leakage spots with the position of the grain boundaries is not possible. If it is assumed that, in (d) and (e), the current is measured basically through GBs (conclusion that can be drawn from the analysis of images c and f), then the measured GB's width is much larger than that in (f). All these effects could be related, as demonstrated in [19], to the contact area increase because of the presence of the water meniscus. Therefore, the results clearly demonstrate that the AFM lateral resolution is very sensitive to the environment, a point that is extremely important when studying polycrystalline high-k dielectrics. Since the grain boundaries width is close to the limit of the AFM resolution, environmental conditions can be the determinant factors to precisely correlate the leakage spots position with the morphological structure of the high-k dielectric.

## Conclusion

The conductivity and charge trapping of amorphous and polycrystalline Al_2_O_3 _layers stacks for memory applications have been studied before and after an electrical stress at nanometer scale using AFM-related techniques in ambient conditions. The current measurements obtained with CAFM before an electrical stress show that the polycrystallization of the Al_2_O_3 _leads to a smaller average and a larger spatial inhomogeneity of the sample conductivity. A statistical analysis of the current images registered on polycrystalline samples has been compared to the measurements obtained with TEM, showing that the mean size of the less conductive areas is similar to the dimensions of the crystals. Therefore, the regions with a smaller conductivity could be related to the grains of the polycrystalline structure: the polycrystals are more insulating whereas the grain boundaries show a larger conductivity. The charge-trapping properties of amorphous and polycrystalline samples were also investigated after an electrical stress. The results suggest that, although the crystals are more resistive and robust (from an electrical point of view) than the amorphous oxide, the grain boundaries of the polycrystalline samples seem to be more sensitive to an electrical stress than those of the non-crystallized structures: grain boundaries would initially act as conductive paths, but would favor a faster charge trapping. Therefore, polycrystallization strongly contributes to the inhomogeneity increase of the conduction and trapping properties of the stacks, which could reduce the reliability of the MOS devices due to the weaker dielectric strength of the grain boundaries. Finally, the influence of the environment conditions on the study of polycrystalline high-k dielectrics was also analyzed. The results demonstrate that the reduction of the water meniscus can be a determinant factor for a precise study in detail on the electrical properties of the grain boundaries.

## Abbreviations

AFM: atomic force microscopy; BD: breakdown; CFAM: conductive atomic force microscope; CPD: contact potential difference; GBs: grain boundaries; KPFM: Kelvin probe force microscope; rms: root mean square; RTP: rapid thermal process; RVS: ramped voltage stresses; TAT: trap-assisted-tunneling; TEM: transmission electron microscopy; UHV: ultra high vacuum.

## Competing interests

The authors declare that they have no competing interests.

## Authors' contributions

ML collected all topographic and current scans performed in air. VI carried out the topographic and current scans performed in ultra high vacuum conditions. MP contributed to the redaction of the manuscript and in the design of the study. MN and XA participated in the design and coordination of the study and reviewed the manuscript. All authors read and approved the final manuscript.
